# The Most Attractive Is Not Always the Preferred: Lessons From Necrophagous Dung Beetle Assemblages in a Region of the Central Amazon

**DOI:** 10.1002/ece3.70766

**Published:** 2024-12-23

**Authors:** Mirella Lima Costa, Renato Portela Salomão, Janderson Batista Rodrigues Alencar, César Murilo de Albuquerque Correa, Luciana Iannuzzi

**Affiliations:** ^1^ Programa de Pós‐Graduação Em Biologia Animal Universidade Federal de Pernambuco Recife Brazil; ^2^ Facultad de Estudios Superiores Iztacala Universidad Nacional Autónoma de México Tlalnepantla de Baz Mexico; ^3^ Pós‐graduação Em Ecologia Instituto Nacional de Pesquisas da Amazônia Manaus Brazil; ^4^ Laboratório de Bioecologia de Scarabaeoidea (Scaralab) Universidade Estadual Do Mato Grosso Do Sul Aquidauana Brazil

**Keywords:** Amazon rainforest, bait preference, carcass, dead corpse, Scarabaeinae, trophic plasticity

## Abstract

Resource attractiveness and preference is determinant to assess how biodiversity is structured in different ecosystems. Necrophagy is the alternative or complementary dietary habit of dung beetles, but a few studies have focused on evaluating how different carrion types attract different species. The goal of this study was to assess the effect of carrion type on attractiveness and preference of dung beetle taxonomic diversity in a region of Central Amazon. Pitfall traps baited with bovine spleen, chicken liver, and fish (freshwater sardine) were installed. Bait attractiveness was estimated through a sampling design that kept exclusively one food type in the field, while bait preference comprised a sampling design with more than one food type offered simultaneously in a concentrated area. We collected 3151 individuals from 24 dung beetle species. Bovine spleen was the most attractive bait in both food preference and food attractiveness experiments, being the only carrion type in which species demonstrated preference. Each carrion type attracted distinct dung beetle assemblages. This study demonstrates that Amazonian dung beetles prefer specific carrion types, which serves as a basis for future studies related to dung beetle diet.

## Graphical abstract

Dung beetles are differently attracted toward carrion types. Bovine spleen attracts more dung beetles than chicken and fish meat.

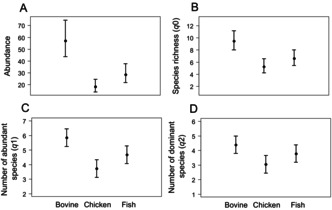

## 1 Introduction

Animals face many choices in their lifetimes, which includes the type of food they eat. Food choice is a complex process that involves recognition and selection, as well as the trade‐offs between the energy invested and gained by using such resources (Schallhart et al. 2012; Latty and Trueblood 2020). Furthermore, food choice reflects how individuals perceive and select food types in their habitats (Agetsuma 1996; Bourg et al. 2016; Salomão, Cerqueira, et al. 2022). Animals are expected to have evolved mechanisms enabling them to choose resources that maximize their fitness (Schallhart et al. 2012; Bailes, Pattrick, and Glover 2018; Latty and Trueblood 2020). For example, honeybee colonies, when presented with a range of options, will choose the most concentrated sugar syrup, up to a threshold concentration (Bailes, Pattrick, and Glover 2018). The assessment of how species are attracted to and prefer various food types may also aid in understanding the spatial coexistence of species (Salomão et al. 2023).

Dung beetles (Coleoptera: Scarabaeidae: Scarabaeinae) are a taxonomically and functionally diverse insect group that primarily consume vertebrate feces; adults use them for feeding and nesting (feeding their offspring, see Halffter and Edmonds 1982; Hanski and Cambefort 1991; Scholtz, Davis, and Kryger 2009). In some regions, such as the Ethiopian, Australian, and especially in the Neotropics, dung beetle species show a wide diversity of feeding habits (Halffter and Halffter 2009). Among the dung beetle diets, necrophagy may complement or substitute coprophagy (Amézquita and Favila 2011; Scholtz, Davis, and Kryger 2009). According to dung beetle evolutionary history, necrophagy is a derived condition from coprophagy (Hanski and Cambefort 1991; Scholtz, Davis, and Kryger 2009). Such derivation is mostly associated with the climate changes that occurred over time, in addition to the decrease in food supply due to the extinction of large vertebrate species between the Pliocene and Pleistocene (5–2 million years) (Halffter and Matthews 1966; Hanski and Cambefort 1991).

Decomposing carcasses (e.g., carrion) are nutritiously rich in microorganisms and bear a doughy texture, which favors their use by a wide array of animals (Dawson et al. 2022; Favila 1993; Selva et al. 2005). Some dung beetles are attracted to fresh and decomposing tissues from dead animals (Halffter and Matthews 1966; Giménez Gómez et al. 2021). While many studies have analyzed dung preference among dung beetle species (e.g., Filgueiras et al. 2009; Bogoni and Hernández 2014), a few studies assessed the attractive potential of carrion types for dung beetles. Most carrion–feeder dung beetles are carrion generalists (Correa et al. 2023); however, there are species that exhibit a degree of specificity/preference to carrion resources (Stavert et al. 2014; Stone, Engasser, and Jameson 2021; Correa et al. 2023).

Carrion is a limited resource both in space and time, which leads to high competition for this food resource (Selva et al. 2005). The high competition for carrion may induce species to rapidly detect and select this decaying food. Dung beetles detect volatile compounds that are released by food resources (e.g., feces, fruits, and carrion), using them as olfactory cues to locate resources (Dormont et al. 2010; Stavert et al. 2014). This fact is critical for carrion search, given that it remains attractive for necrophagous beetles for longer periods (up to 1 month; see Mayer and Vasconcelos 2013). Moreover, carrion is widely used by vertebrates (Lira, Aguiar Silvieira, and Frizzas 2020), which may consume the whole carcass rapidly in natural conditions (DeVault, Rhodes Jr., and Shivik 2003; Hill et al. 2018). Thus, the potential of carrion types to attract dung beetles can be an important indicator food usage by these insects. The simultaneous availability of different carrions allows us to understand whether necrophagous dung beetles prefer specific carrion types (Stavert et al. 2014; Correa et al. 2023). When analyzing dung beetle diet, food attractiveness and food preference experiments can present important insights about diet plasticity. While food attractiveness experiments allow the assessment of the of a food type to attract dung beetles—independent from choice of foods—food preference experiments tested the preference of food types offered together (adapted from Filgueiras et al. 2009; Bogoni and Hernández 2014; Salomão and Iannuzzi 2017; Salomão et al. 2023). The goal of this study was to assess the effect of bait attractiveness and preference on dung beetle taxonomic diversity in a region of Central Amazon. We tested the effect of three carrion types (chicken liver, bovine spleen, and freshwater sardine—hereafter “fish”) on dung beetle taxonomic diversity, abundance, and assemblage structure (i.e., the distribution of species and their abundances). We also tested whether species presented a preference toward one of the carrion types. Based on previous data (Stavert et al. 2014; Iannuzzi et al. 2016), we expect that a higher diversity and abundance of dung beetles will be attracted to and prefer bovine spleen when compared to chicken liver and fish.

## 2 Materials and Methods

### 2.1 Study Site

We carried out this study in Adolpho Ducke's Forest Reserve (2°57′′ S, 59°55′ W, ca. 90 m a. s. l.), located in Manaus, Amazonas state, Brazil. This reserve is directed by Instituto Nacional de Pesquisas da Amazônia and contains approximately 10,000 ha of conserved area, with minimal disturbance levels. Vegetation physiognomy is composed mostly by ombrophilous nonfloodable forest (*terra firme*) and floodable forests (*igapós*). The climate of the region is classified as tropical, hot, and humid (Af) according to Köppen's classification and has a mean annual temperature of 26°C, ranging between 19°C and 39°C throughout the year (2022). The region has a mean annual rainfall ranging between 1900 and 2300 mm, with two marked seasons—a rainy season between December and May (mean monthly rainfall: 277.76 mm) and a dry season between June and November (mean monthly rainfall: 78.93 mm) (Ferreira et al. 2012; INMET 2022).

Surrounding the studied reserve there are two large blocks of land‐cover types: (1) an urban landscape, comprising Manaus, one of the largest cities in the Amazon region; and (2) a conserved forest continuum. The experiment was performed in conserved forests at least 3‐km distant from the direct urban influence of the city of Manaus and other human establishments (e.g., villages). As this reserve has never been deforested due to urban expansion or any Western practices, such as cattle ranching, mineral extraction, or selective logging, this study site serves as a good model to assess ecological patterns of native ecosystems.

### 2.2 Food Attractiveness and Food Preference Experiments

Dung beetles were sampled in July 2022, comprising the dry season of the region (Baccaro et al. 2008). Such season in this Amazonian region is marked by a high activity of dung beetles (Ratcliffe 2013), allowing adequate representativeness of their assemblages. For each experiment (food attractiveness and food preference), pitfall traps were installed in linear transects in the field to capture dung beetles. These pitfall traps were baited with different carrion types (bovine spleen, chicken liver, and fish), which had been allowed to decompose in sealed containers for 48 h prior to the experiment. The carrion types selected have been effective in previous ecological studies of dung beetles in neotropical region (Mora‐Aguilar et al. 2023). Although dung beetles do not consume fish meat underwater, inundation pools often lead to dead fishes in riverbanks in tropical rainforests (Chapman and Kramer 1991; Junk et al. 2020), and vertebrates that prey on fishes often leave their leftovers on the forest surface (*personal observation*). Therefore, dead fishes represent a potential carrion type for dung beetles in natural conditions. Although it is unlikely that bovine carrion represents a common food resource in Amazonian forests, such carrion could resemble native mammal carrion, as jaguars ( *Panthera onca* ), tapirs ( *Tapirus terrestris* ), sloths (*Bradypus* spp.), agoutis (*Dasyprocta* spp.), and pacas ( *Cuniculus paca* ). The same rationale was used to select chicken liver for this research.

Each pitfall trap consisted of a 1000‐mL cylindrical plastic container (13 cm high and 15 cm diameter), buried at the soil surface. Above the pitfall trap, ca. 25 g of carrion was deposited in a metal cage, to avoid being stolen by other animals. Inside pitfall traps, a 200 mL solution of water, salt (ca. 5 g), and odor‐free detergent (ca. 5 mL) was used to preserve captured dung beetles and prevent their escape. A plastic lid was secured over the trap to prevent the entry of rainwater and debris. After a 48‐h period, dung beetles that had fallen into the traps were collected and stored in plastic vials containing 70% ethanol.

Samplings were performed for two experiments: bait attractiveness and bait preference. Bait attractiveness was estimated by a sampling design that allowed exclusively one food type in the field, while bait preference comprised a sampling design with more than one food type offered simultaneously in a concentrated area‐restricted space (Salomão et al. 2023). Attractiveness experiment tested the attractive potential of each carrion type (i.e., only one carrion type) toward dung beetles. This setup minimized potential bias from the attractive effects that might have been caused by the availability of other neighboring food types. The experiment of preference investigated which types of carrion dung beetles prefer when multiple options are available.

The attractiveness experiment comprised 15 sets of pitfall traps for each carrion type, arranged in linear transects. Each set consisted of two pitfall traps, both baited with the same type of carrion and spaced 5 m apart. In the setup, pitfall sets containing the same carrion type were installed sequentially, while sets with different carrion types were spaced at least 150 m apart to minimize interference (Figure S1). In total, the experiment involved 45 replicates, consisting of 15 for each of the three bait types, with sampling effort encompassed 90 pitfall traps (15 replicates × 2 pitfall traps × 3 treatments).

The carrion preference experiment comprised 15 sets of pitfall traps, each set comprising the three carrion types simultaneously. Since this experiment comprised a sampling design with different bait types offered simultaneously and near each other, control traps (without bait) were used to test whether dung beetles could randomly fall in any of the traps. Each set comprised eight pitfall traps (two for each carrion type and two control traps), installed 2 m apart from one another (Figure S2). In attractiveness and preference experiments, pitfall trap sets were spaced at least 150 m apart to ensure spatial independence among samples (Larsen and Forsyth 2005; Silva and Hernández 2015). The carrion preference experiment had a total effort of 60 replicates, 15 for each carrion type and 15 for the control (15 replicates × 2 pitfall traps × 4 treatments [3 bait types and a control] = 120 pitfall traps).

The collected material was identified to species level by using literature (Génier 2009; González‐Alvarado and Vaz‐de‐Mello 2021) and by comparisons with deposited specimens in the Entomological Collection of the Instituto Nacional de Pesquisas da Amazônia (Manaus, Amazonas, Brazil). The specimens were subsequently deposited in the Entomological Collection of Universidade Federal de Pernambuco (CEUFPE, Recife, Pernambuco, Brazil).

### 2.3 Data Analyses

To estimate the completeness of dung beetle diversity in each experiment, we performed Sample Coverage estimation in iNEXT online software (Hsieh, Ma, and Chao 2016). This estimation considers the species richness, relative abundance, and the rare species in the samples. We performed Sample Coverage for each bait type.

We estimated taxonomic diversity of dung beetles collected in each bait type and in each experiment by calculating the Hill numbers (Jost 2006). Diversity numbers were estimated in the orders *q* = 0 (*q*0, species richness, which does not account species abundance), *q* = 1 (*q*1, exponential of Shannon entropy, which is sensitive to species abundance and is a proxy of the number of abundant species in a sample), and *q* = 2 (*q*2, inverse of Simpson, which gives higher weight to species abundance than *q*1 and indicates the number of dominant species) (Hill 1973; Jost 2006). The estimation of diversity numbers was performed in iNEXT online software (Chao and Chiu 2016; Hsieh, Ma, and Chao 2016).

To assess the effect of carrion type in each experiment (food preference and food attractiveness) on dung beetles' diversity numbers and abundance, we performed generalized linear models (GLMs). Control traps were used in food preference GLMs. For species richness (*q*0) data, we used Poisson error distribution, which is a proper distribution family for these data (Zuur et al. 2009). Due to the overdispersion in abundance data (Residual deviance/Residual d. f. > 2), we used negative binomial error distribution. For the number of abundant (*q*1) and dominant species (*q*2), we used Gaussian distribution, which was the model that best fitted these data. We tested homoscedasticity of the GLMs by using the Fligner–Killeen test (Fligner and Killeen 1976). We checked data distribution visually by using q‐q plots, and we used Cook's distance to test the presence of outliers (Cooks' Distance > 1.0). In the food preference experiment, taxonomic diversity data were log‐transformed to improve models fit. Data were analyzed using R software version 4.1.2 (R Development Core Team 2022).

To assess more precisely the attraction of dung beetle species to various carrion types, we applied the Indicator Value (IndVal) method. This analysis combines specificity degree, estimated through relative abundances, and fidelity degree, estimated by the degree of incidence—both specificity and fidelity degree were assessed relative to the carrion types (Dufrêne and Legendre 1997; McGeoch, Van Rensburg, and Botes 2002). For a broader comprehension and clearer biological meaning of carrion specificity among dung beetle species, we used data from both experiments together. We performed IndVal in labdsv package (Cáceres and Legendre 2009) in R software (R Development Core Team 2022).

To test whether carrion types affected the dung beetle assemblage structure (i.e., species composition considering its abundances) in each experiment, we performed permutational analysis of variances (PERMANOVA). Moreover, we performed permutational analysis of dispersion (PERMDISP) to statistically compare the multivariate dispersion of dung beetle assemblage structure in each carrion type. For PERMANOVA, we used 9999 randomizations, while for PERMDISP, we performed 999 random events (Anderson, Ellingsen, and McArdle 2006). We used post hoc Tukey's test to compare dispersion (PERMDISP) observed of assemblage structure data recorded in each carrion type. Both PERMANOVA and PERMDISP were ran in vegan package (Oksanen et al. 2020), in R software (R Development Core Team 2022). For PERMANOVA and PERMDISP, we used similarity matrices obtained from the Bray–Curtis similarity index.

## 3 Results

### 3.1 Species Diversity

A total of 3151 individuals were collected, 3145 in baited traps and six in control traps, belonging to 13 genera and 24 species (Table 1). Three species were highly abundant: *Deltochilum* species‐group *submetalicum* and *Deltochilum* species‐group *aspericolle* and *Canthon triangularis*, comprising together approx. 60% of the individuals collected. Fourteen species comprised, each one, less than 1% of relative abundance (Table 1). Two species were singletons—*Onthophagus* aff. *osculatti* and *Oxysternon durantoni*. Regarding data distribution among carrion types in both experiments, 1560 individuals identified in 22 species were collected in pitfall traps baited with bovine spleen; 837 individuals identified in 19 species were collected in fish‐baited pitfall traps; 748 individuals from 18 species were collected in chicken‐baited pitfall traps. Sample coverage ranged between 98% and 100% (see Table 1), indicating that we had an appropriate effort to represent the dung beetle assemblages in our study.

**TABLE 1:** Abundance, species richness, and resource preference (Indicator Value—IndVal) of dung beetle species collected through pitfalls baited with bovine spleen, chicken liver, and fish. Data obtained from food preference and attractiveness experiments, in the Adolpho Ducke Forest Reserve, Amazonas, Brazil. A—Food attractiveness experiment and P—food preference experiment. Species with *n* < 5 individuals were considered “too rare.”

Taxa	Carrion types						IndVal	Total
	Bovine spleen		Chicken liver		Fish			
	A	P	A	P	A	P		
Ateuchini								
*Ateuchus globulus* (Boucomont, 1928)	1	0	0	2	1	4	Generalist	8
*Ateuchus simplex* (Le Peletier & Serville, 1828)	0	5	0	0	0	0	Specialist	5
*Eutrichillum* sp.	0	1	1	0	3	1	Generalist	6
Deltochilini								
*Canthon quadriguttatus* (Olivier, 1789)	1	0	1	2	0	1	Generalist	5
*Canthon sordidus* (Harold, 1868)	31	31	51	17	22	7	Generalist	159
*Canthon triangularis* (Drury, 1770)	130	141	8	77	13	40	Specialist	409
*Deltochilum* species‐group *aspericolle* Bates, 1870	54	160	17	106	111	136	Generalist	584
*Deltochilum carinatum* (Westwood, 1837)	10	4	3	2	1	4	Generalist	24
*Deltochilum icarus* (Olivier, 1789)	2	3	1	6	2	1	Generalist	15
*Deltochilum* species‐group *sextuberculatum* (Bates, 1870)	0	0	1	0	1	1	Too rare	3
*Deltochilum* species‐group *submetallicum* (Castelnau, 1840)	347	187	76	147	132	127	Specialist	1016
*Scybalocanthon pygidialis* (Schmidt, 1922)	3	1	0	0	0	0	Too rare	4
*Hansreia coriacea* (Schmidt, 1922)	0	0	5	0	0	0	Generalist	5
Dichotomini								
*Dichotomius lucasi* (Harold, 1869)	24	10	23	8	5	5	Generalist	75
*Dichotomius boreus* (Olivier, 1789)	2	2	4	0	2	1	Generalist	11
Oniticellini								
*Eurysternus atrosericus* (Génier 2009)	27	12	0	5	7	7	Specialist	58
*Eurysternus caribaeus* (Herbst, 1789)	71	20	8	8	2	2	Specialist	111
Onthophagini								
*Onthophagus* aff. *osculatii* (Guérin‐Méneville, 1855)	0	1	0	0	0	0	Too rare	1
Phanaeini								
*Coprophanaeus jasius* (Olivier, 1789)	66	16	62	52	45	26	Generalist	267
*Coprophanaeus lancifer* (Linnaeus, 1767)	44	12	14	28	20	27	Generalist	145
*Oxysternon festivum* (Linnaeus, 1758)	1	1	0	0	1	0	Too rare	3
*Oxysternon durantoni* (Arnaud, 1984)	1	0	0	0	0	0	Too rare	1
Genera *incertae sedis* in Scarabaeinae								
*Canthidium* sp.	41	97	1	12	61	17	Specialist	229
*Uroxys* sp.	1	0	0	0	1	0	Too rare	2
Number of individuals (abundance)	856	704	276	472	430	407		3145
Number of species (Species richness)	19	18	16	14	18	17		102
Sampling coverage (%)	99.55%	99.41%	98.27%	100%	99.13%	99.02%		

**FIGURE 1:**
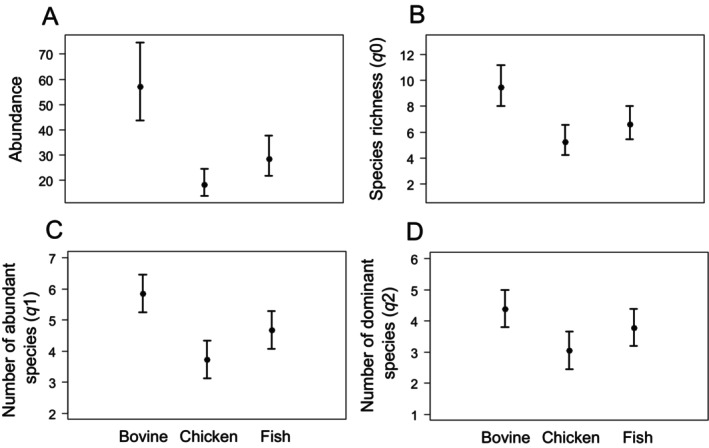
Food attractiveness experiment, presenting abundance, and taxonomic diversity (Hill numbers) of dung beetle collected through pitfalls baited with bovine spleen, chicken liver, and fish in Adolpho Ducke Forest Reserve, Amazonas, Brazil.

For the experiment of food attractiveness, a total of 1562 individuals from 22 species were recorded. Pitfall traps baited with bovine spleen captured most individuals (*n* = 856) and species (*s* = 19), followed by fish‐baited traps (*n* = 430, *s* = 18). Pitfall traps baited with chicken liver captured the lowest abundance and species richness of dung beetles in the food attractiveness experiment (*n* = 276, *s* = 16). There was a higher dung beetle abundance recorded in bovine‐spleen pitfall traps compared to chicken and fish ones (*χ* ^2^_2,42_ = 47.32, *p* < 0.01, Figure 1A). Species richness (*q*0) (*χ* ^2^_2,42_ = 18.08, *p* < 0.01) and the number of abundant species (*q*1) (*χ* ^2^_2,42_ = 56.59, *p* < 0.01) were higher in bovine‐spleen baited traps than the other carrion types (Figure 1B,C). The number of dominant species (*q*2) (*χ* ^2^_2,42_ = 55.17, *p* < 0.01) was higher in bovine‐spleen traps compared to chicken‐baited traps (Figure 1D).

**FIGURE 2:**
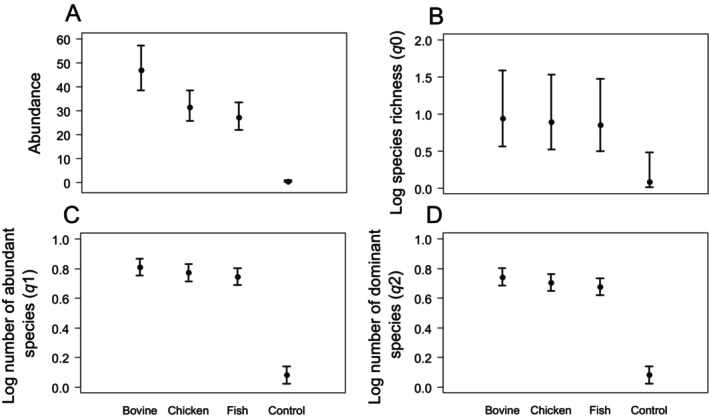
Food preference experiment, presenting abundance, and taxonomic diversity (Hill numbers) of dung beetle collected through pitfalls baited with bovine spleen, chicken liver, and fish in Adolpho Ducke Forest Reserve, Amazonas, Brazil.

For the food preference experiment, 1583 individuals from 21 species were collected. Bovine‐spleen baited traps recorded a total of 704 individuals from 18 species and chicken‐liver baited traps recorded 472 individuals from 14 species, while fish‐baited pitfall traps recorded a total of 407 individuals from 17 species. For the control traps, six individuals of five species were collected: *Canthidium* sp. (*n* = 1), *D*. species‐group *aspericolle* (*n* = 1), *Coprophanaeus jasius* (*n* = 1), *Ateuchus globulus* (*n* = 2), and *D*. species‐group *submetallicum* (*n* = 1). In food preference experiment, there was a significantly lower abundance (*χ* ^2^_3,56_ = 64.07, *p* < 0.01), species richness (*χ* ^2^_3,56_ = 4.48, *p* < 0.01), number of abundant (*χ* ^2^_3,56_ = 0.70, *p* < 0.01), and dominant species (*χ* ^2^_3,56_ = 0.71, *p* < 0.01) in control traps than carrion baited traps. Bovine carrion attracted more individuals than other carrion types; however, no difference in taxonomic diversity was observed among carrion types (Figure 2).

**FIGURE 3:**
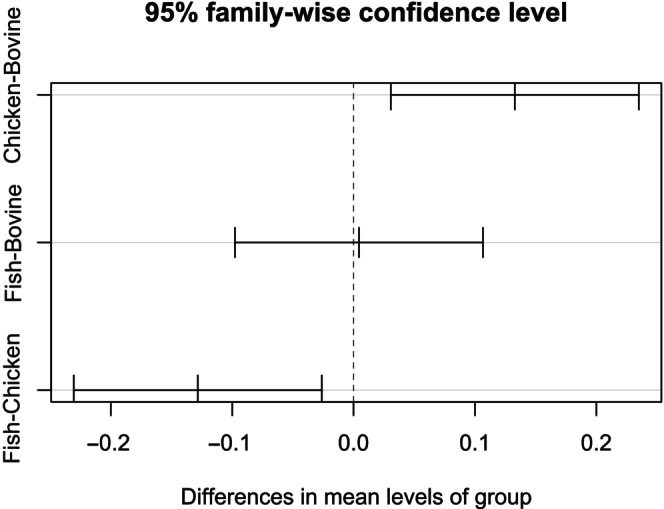
Tukey's post hoc test comparing dispersion patterns of samples between bait types for the food attractiveness experiment. Traps baited with chicken liver showed greater data dispersion.

Despite the small number of specimens, *Ateuchus simplex*, *O*. aff. *osculatti*, *Scybalocanthon pygidialis*, and *O. durantoni* were collected exclusively in bovine‐spleen baited traps, and *Hansreia coriacea* was exclusively recorded in chicken liver and fish‐baited traps (Table 1). Six species were significantly associated with bovine‐spleen baits: *Canthidium* sp. (IndVal = 0.52, *p* < 0.01), *Canthon triangularis* (IndVal = 0.64, *p* < 0.01), *D*. species‐group *submetalicum* (IndVal = 0.51, *p* < 0.01), *Eurysternus atrosericus* (IndVal = 0.38, *p* < 0.01), *A. simplex* (IndVal = 0.13, *p* = 0.03), and *Eurysternus caribaeus* (IndVal = 0.63, *p* < 0.01) (Table 1). No species were significantly associated with the other carrion types. According to PERMANOVA, carrion types structured different dung beetle assemblages, both in food attractiveness (*F* = 6.64; *p* < 0.01) and food preference experiments (*F* = 3.82, *p* < 0.01). However, in the food preference experiment, there was no difference between chicken and fish (Table S1). Data dispersion was distinct among carrion types in food attractiveness experiment (PERMDISP: *F* = 6.43, *p* < 0.01), with chicken‐liver pitfall traps presenting a higher data dispersion (Chicken = 40.04 ± 2.62) when compared to fish‐baited (Fish = 27.66 ± 2.65) and bovine‐baited (Bovine = 22.57 ± 1.37) pitfall traps (Figure 3). There was no difference in data dispersion of carrion types in food preference experiment (PERMDISP: *F* = 0.65, *p* = 0.52).

## 4 Discussion

Dung beetles have an extensive range of feeding habitats, from highly specific dietary preferences to more generalist ones (Vaz‐De‐Mello 2008; Halffter and Halffter 2009; Costa et al. 2013; Salomão and Iannuzzi 2017). A few studies have assessed the effect of carrion types in food attractivity and preference of dung beetles (e.g., Stavert et al. 2014; Stone, Engasser, and Jameson 2021; Correa et al. 2023). The current study deepens the comprehension regarding necrophagous diet of Amazonian dung beetles. Carrion types attracted different assemblages, with bovine spleen capturing a higher diversity than chicken liver and fish. Moreover, bovine spleen was the only carrion type in which species showed a high fidelity, partially confirming our prediction—that bovine spleen would be more attractive (although not the most preferred), than chicken liver and fish. Carrion is often used only as a complementary bait type in ecological studies of dung beetle assemblages (Ebert et al. 2019; Mora‐Aguilar et al. 2023; Salomão et al. 2023). The results presented herein allowed us to expand our knowledge of food recognition among necrophagous dung beetles and better understand species distribution among different carrion types in Amazonian forests.

Although bovine spleen attracted a higher taxonomic diversity (i.e., food attractiveness experiment), at assemblage scale, it was not the most preferred (i.e., food preference experiment). However, some species demonstrated a preference for bovine spleen, among them *E. atrosericus* and *E. caribaeus*. These species have been reported in bovine carrion (see Correa et al. 2023); while *E. atrosericus* has been reported only in cattle beef, *E. caribaeus* was reported in different parts of bovine carrion, such as muscle and liver. Our results, together with the above‐mentioned study, suggest that these species—despite feeding mainly on dung (see Salomão et al. 2023; Puker et al. 2024)—have the capacity to distinguish between carrion types, which allows them to locate and exploit carrion in tropical forests. Higher diversities of dung beetles in a spatiotemporal and trophic resource context are often related to a broader resource usage in space and time and food sources (Liberal et al. 2011; Salomão and Iannuzzi 2017; Salomão, Alcântra Pires, et al. 2022; Salomão, Cerqueira, et al. 2022; Correa et al. 2021). The high diversity observed in bovine spleen could suggest that this carrion type could allow the coutilization of this food type by of a higher number of species when compared to the other carrions. There is fierce competition for food among dung beetles (Hanski and Cambefort 1991; Scholtz, Davis, and Kryger 2009). As the quality of the resources can affect the fitness of the individuals (Favila 2001), dung beetles could prefer high‐quality resources (but see Frank et al. 2017), and thus, carrion quality could be affecting the diversity patterns observed herein. For example, the necrophagous dung beetle *Canthon cyanellus* can breed on different types of carrion, low‐quality food, such as carrion with high proportion of lipids and low proportion of proteins, led to a reduced parental fitness (Favila 2001). Besides food quality, more abundant food sources tend to attract a higher diversity of dung beetles (Finn and Giller 2000). Tropical forests harbor small‐ and medium‐sized mammals, which are larger than most birds and fish. Food recognition in dung beetles is mostly driven by olfactory cues (Hanski and Cambefort 1991; Scholtz, Davis, and Kryger 2009). In this sense, the preference for specific food types depends on the volatile compounds produced by the food resource (Hanski and Cambefort 1991; Holter and Scholtz 2007; Bogoni and Hernandez, Bogoni and Hernández 2014). Dimethyl trisulfate is a notable volatile compound produced by bovine carrion (Stavert et al. 2014). One hypothesis is that mammalian carrion emits similar volatile compounds, which could uniformly attract dung beetles. By examining the behavioral traits of the dung beetle species that recognize and are attracted to volatiles produced by different vertebrate carrion types, we can gain more precise insights into how bovine carrion volatiles influence dung beetle diversity.

Interestingly, in the food attraction experiment, traps baited with chicken liver exhibited greater data dispersion compared to those baited with other carrion types. Although ecological data are commonly asymmetrically distributed (Greenacre 2016), dung beetles attracted to bovine spleen and fish meat showed a more homogenous assemblage structure. The multivariate approach used in this study served as a proxy of beta diversity (Anderson, Ellingsen, and McArdle 2006) and suggested that chicken liver recorded more unstable (heterogeneous) dung beetle assemblages. Similarly, studies on saproxylic beetles in Central Amazonia and canopy beetles in tropical forests of Australia have both found that resource heterogeneity within tropical trees plays a crucial role in maintaining beta diversity (Alencar et al. 2021; Wardhaugh, Stork, and Edwards 2012). A previous study with *Dichotomius* beetles from Atlantic forest of Brazil suggested that food recognition mediated by olfactory cues could depend on individual traits, as sex and age (Salomão, Cerqueira, et al. 2022). We hypothesize that bovine spleen and fish provide dependable and stable olfactory cues for dung beetles, while the attractiveness of chicken liver may be dependent on individual traits and environmental variables (e.g., availability of different resources, air humidity, temperature), thus leading to more heterogeneous dung beetle assemblages.

When comparing the results found between food attractiveness and food preference, diversity was markedly different between food types in food attractiveness—with bovine spleen recording higher diversity than the other food types—but not in food preference experiment. The differences observed between experiments can be related to the energy demanded by dung beetles for foraging activities, the confounding factor that could be acting on dung beetle perception of food types, and the selectiveness whenever different food types are simultaneously offered. Carrion and dung are randomly distributed in the ecosystems, and finding and removing these food resources is energetically costly for dung beetles (Heinrich and Bartholomew 1979; Cambefort and Hanski 1991; Krell et al. 2003; Scholtz, Davis, and Kryger 2009). Albeit food choice can be strict in some scenarios (e.g., Salomão and Iannuzzi 2017; Salomão et al. 2023), confounding factors may also affect the selection of food types by dung beetles. For example, dung beetles may find misleading odor cues that lead them to interact and pollinize *Orchidantha* brown flowers in Malaysia, although not using them as a food resource (Sakai and Inoue 1999). We hypothesize that, under the condition of specific food types that are available and spaced from other food types (i.e., food attraction experiment), the energetical investment to find them will lead to a more marked food choice when compared to scenarios where different food types are simultaneously offered (i.e., food choice experiment). This would lead to clear differences in dung beetle diversity and assemblage structure between food types in food attraction experiments. Thus, when different food types of a similar kind of resource are close, olfactory cues could lead to a random selection of food, masking the differences between them and leading to similar dung beetle assemblages among carrion types. In this sense, the food preference experiment should be analyzed with care—the patterns presented could suffer from a sampling bias where carrion spacing (2 m among carrion types) would not allow dung beetles to proper detect odor plumes and select their preferred carrion. Nonetheless, it is important to consider that such 2‐m trap spacing is recommended in the literature to evaluate food preference (see Louzada and Silva 2009; Correa et al. 2018; Mora‐Aguilar et al. 2023). Considering that necrophagy is relatively recent in Scarabaeinae beetles (Halffter and Matthews 1966; Hanski and Cambefort 1991), speciation processes to use the different carrion types by such beetles are still in progress (Gillet and Toussaint 2020). This could lead to a potential lack of carrion speciation among the most dung beetle species, resulting in an absence of carrion preference at assemblage level.

## 5 Conclusions

In synthesis, this study highlighted the role of different carrion types in the segregation of dung beetle assemblages in Amazonian forests. Like studies that demonstrated the importance of different dung types for the dung beetle assemblage structure in other tropical rainforests (Filgueiras et al. 2009; Bogoni and Hernández 2014), here we demonstrate that dung beetle species can present preferences toward specific carrion types—although such preferences can depend on the spatial scale of food availability. Our study presented broad but still superficial patterns, which may be deepened by future finer assessment of the mechanisms that drives carrion choice by dung beetle species. Volatile dispersion, arena observations, physiological, and electroantennogram experiments can be future study fields that aid in deepening the knowledge of carrion perception and use by dung beetles.

## Author Contributions

**Mirella Lima Costa:** conceptualization (equal), data curation (equal), formal analysis (equal), investigation (equal), writing – original draft (equal), writing – review and editing (equal). **Renato Portela Salomão:** conceptualization (equal), data curation (equal), formal analysis (equal), funding acquisition (equal), investigation (equal), methodology (equal), project administration (equal), resources (equal), supervision (equal), validation (equal), visualization (equal), writing – original draft (equal), writing – review and editing (equal). **Janderson Batista Rodrigues Alencar:** data curation (equal), investigation (equal), methodology (equal), validation (equal), writing – review and editing (equal). **César Murilo de Albuquerque Correa:** data curation (equal), formal analysis (equal), supervision (equal), writing – original draft (equal), writing – review and editing (equal). **Luciana Iannuzzi:** conceptualization (equal), data curation (equal), funding acquisition (equal), investigation (equal), methodology (equal), project administration (equal), supervision (equal), visualization (equal), writing – original draft (equal), writing – review and editing (equal).

## Conflicts of Interest

The authors declare no conflicts of interest.

## Supporting information


Data S1.

## Data Availability

All data are presented as Supporting Information.

## References

[ece370766-bib-0001] Agetsuma, N. 1996. “Dietary Selection by Yakushima Macaques ( *Macaca fuscata yakui* ): The Influence of Food Availability and Temperature.” International Journal of Primatology 16: 611–627.

[ece370766-bib-0002] Alencar, J. B. R. , C. R. V. Fonseca , D. M. Marra , and F. B. Baccaro . 2021. “Windthrows Promote Higher Diversity of Saproxylic Beetles (Coleoptera: Passalidae) in a Central Amazon Forest.” Insect Conservation and Diversity 15: 1–8.

[ece370766-bib-0003] Amézquita, S. , and M. E. Favila . 2011. “Carrion Removal Rates and Diel Activity of Necrophagous Beetles (Coleoptera: Scarabaeinae) in a Fragmented Tropical Rain Forest.” Environmental Entomology 40: 239–246. 10.1603/EN09182 20388260

[ece370766-bib-0004] Anderson, M. J. , K. E. Ellingsen , and B. H. McArdle . 2006. “Multivariate Dispersion as a Measure of Beta Diversity.” Ecology Letters 9: 683–693. 16706913 10.1111/j.1461-0248.2006.00926.x

[ece370766-bib-0005] Baccaro, F. B. , D. P. Drucker , J. D. Vale , et al. 2008. A Reserva Ducke. Reserva Ducke, A Biodiversidade Amazônica Através de Uma Grade, edited by M. L. Oliveira , F. B. Baccaro , R. Braga‐Neto , and W. E. Magnusson , 166. Manaus, Brazil: Editora INPA.

[ece370766-bib-0006] Bailes, E. J. , J. G. Pattrick , and B. J. Glover . 2018. “An Analysis of the Energetic Reward Offered by Field Bean ( *Vicia faba* ) Flowers: Nectar, Pollen, and Operative Force.” Ecology and Evolution 8: 3161–3171. 29607015 10.1002/ece3.3851 PMC5869266

[ece370766-bib-0007] Bogoni, J. A. , and M. I. Hernández . 2014. “Attractiveness of Native Mammal's Feces of Different Trophic Guilds to Dung Beetles (Coleoptera: Scarabaeinae).” Journal of Insect Science 14: 299. 25528749 10.1093/jisesa/ieu161 PMC5657881

[ece370766-bib-0008] Bourg, A. , F. Escobar , I. MacGregor‐Fors , and C. E. Moreno . 2016. “Got Dung? Resource Selection by Dung Beetles in Neotropical Forest Fragments and Cattle Pastures.” Neotropical Entomology 45: 490–498. 27147229 10.1007/s13744-016-0397-7

[ece370766-bib-0009] Cáceres, M. D. , and P. Legendre . 2009. “Associations Between Species and Groups of Sites: Indices and Statistical Inference.” Ecology 90: 3566–3574. 20120823 10.1890/08-1823.1

[ece370766-bib-0010] Cambefort, Y. , and I. Hanski . 1991. “Dung Beetle Population Biology.” Dung Beetle Ecology 1: 36–50.

[ece370766-bib-0011] Chao, A. , and C. H. Chiu . 2016. “Species Richness: Estimation and Comparison.” In Wiley StatsRef: Statistics Reference Online, edited by N. Balakrishnan , T. Colton , B. Everitt , et al., vol. 1, 26. Hoboken, USA: John Wiley & Sons.

[ece370766-bib-0012] Chapman, L. J. , and D. L. Kramer . 1991. “The Consequences of Flooding for the Dispersal and Fate of Poeciliid Fish in an Intermittent Tropical Stream.” Oecologia 87: 299–306. 28313849 10.1007/BF00325270

[ece370766-bib-0013] Correa, C. M. , K. R. Ferreira , A. Puker , L. D. Audino , and V. Korasaki . 2021. “Greenspace Sites Conserve Taxonomic and Functional Diversity of Dung Beetles in an Urbanized Landscape in the Brazilian Cerrado.” Urban Ecosystems 24: 1023–1034.

[ece370766-bib-0014] Correa, C. M. , R. P. Salomão , B. F. Xavier , A. Puker , and K. R. Ferreira . 2023. “Not All Dung Beetles Feed on Dung: Scarabaeinae (Coleoptera: Scarabaeidae) Attracted to Different Carrion Types in Contrasting Habitats at Brazilian Amazon.” Austral Ecology 48: 952–968.

[ece370766-bib-0015] Correa, C. M. A. , R. F. Braga , A. Puker , A. R. Abot , and V. Korasaki . 2018. “Optimising Methods for Dung Beetle (Coleoptera: Scarabaeidae) Sampling in Brazilian Pastures.” Environmental Entomology 47: 48–54. 29293908 10.1093/ee/nvx191

[ece370766-bib-0016] Costa, F. C. , K. K. Pessoa , C. N. Liberal , B. K. Filgueiras , R. P. Salomão , and L. Iannuzzi . 2013. “What Is the Importance of Open Habitat in a Predominantly Closed Forest Area to the Dung Beetle (Coleoptera, Scarabaeinae) Assemblage?” Revista Brasileira de Entomologia 57: 329–334.

[ece370766-bib-0017] Dawson, B. M. , J. F. Wallman , M. J. Evans , and P. S. Barton . 2022. “Insect Abundance Patterns on Vertebrate Remains Reveal Carrion Resource Quality Variation.” Oecologia 198: 1043–1056. 35294646 10.1007/s00442-022-05145-4 PMC9056491

[ece370766-bib-0018] DeVault, T. L. , O. E. Rhodes Jr. , and J. A. Shivik . 2003. “Scavenging by Vertebrates: Behavioral, Ecological, and Evolutionary Perspectives on an Important Energy Transfer Pathway in Terrestrial Ecosystems.” Oikos 102: 225–234.

[ece370766-bib-0019] Dormont, L. , P. Jay‐Robert , J. M. Bessière , S. Rapior , and J. P. Lumaret . 2010. “Innate Olfactory Preferences in Dung Beetles.” Journal of Experimental Biology 213: 177–3186. 10.1242/jeb.040964 20802120

[ece370766-bib-0020] Dufrêne, M. , and P. Legendre . 1997. “Species Assemblages and Indicator Species: The Need for a Flexible Asymmetrical Approach.” Ecological Monographs 67: 345–366.

[ece370766-bib-0021] Ebert, K. M. , G. B. Monteith , R. Menéndez , and D. J. Merritt . 2019. “Bait Preferences of Australian Dung Beetles (Coleoptera: Scarabaeidae) in Tropical and Subtropical Queensland Forests.” Austral Entomology 58: 772–782.

[ece370766-bib-0023] Favila, M. E. 1993. “Some Ecological Factors Affecting the Life‐Style of *Canthon cyanellus* (Coleptera Scarabaeidae): An Experimental Approach.” Ethology Ecology and Evolution 5: 319–328.

[ece370766-bib-0024] Favila, M. E. 2001. “Historia de Vida y Comportamiento de un Escarabajo Necrófago: * Canthon cyanellus cyanellus* LeConte (Coleoptera: Scarabaeinae).” Folia Entomológica Mexicana 40: 245–278.

[ece370766-bib-0025] Ferreira, S. J. F. , S. Á. F. Miranda , A. D. O. Marques Filho , and C. C. Silva . 2012. “Efeito da Pressão Antrópica Sobre Igarapés na Reserva Florestal Adolpho Ducke, área de Floresta na Amazônia Central.” Acta Amazonica 42: 533–540.

[ece370766-bib-0026] Filgueiras, B. K. , C. N. Liberal , C. D. Aguiar , M. I. M. Hernández , and L. Iannuzzi . 2009. “Attractivity of Omnivore, Carnivore and Herbivore Mammalian Dung to Scarabaeinae (Coleoptera, Scarabaeidae) in a Tropical Atlantic Rainforest Remnant.” Revista Brasileira de Entomologia 53: 422–427.

[ece370766-bib-0027] Finn, J. A. , and P. S. Giller . 2000. “Patch Size and Colonisation Patterns: An Experimental Analysis Using North Temperate Coprophagous Dung Beetles.” Ecography 23: 315–327.

[ece370766-bib-0028] Fligner, M. A. , and T. J. Killeen . 1976. “Distribution‐Free Two‐Sample Tests for Scale.” Journal of the American Statistical Association 71: 210–213.

[ece370766-bib-0029] Frank, K. , A. Brückner , A. Hilpert , M. Heethoff , and N. Blüthgen . 2017. “Nutrient Quality of Vertebrate Dung as a Diet for Dung Beetles.” Scientific Reports 7: 12141. 28939910 10.1038/s41598-017-12265-y PMC5610319

[ece370766-bib-0030] Génier, F. 2009. Le genre Eurysternus Dalman, 1824 (Scarabaeidae: Scarabaeinae: Oniticellini): Révision Taxonomique et clés de Détermination Illustrées. Vol. 85, 1–432. Sofia, Bulgaria: Pensoft.

[ece370766-bib-0031] Gillet, C. P. D. T. , and E. F. A. Toussaint . 2020. “Macroevolution and Shifts in the Feeding Biology of the New World Scarab Beetle Tribe Phanaeini (Coleoptera: Scarabaeidae: Scarabaeinae).” Biological Journal of the Linnean Society 130: 661–682.

[ece370766-bib-0032] Giménez Gómez, V. C. , J. R. Verdú , S. J. E. Velazco , and G. A. Zurita . 2021. “Dung Beetle Trophic Ecology: Are We Misunderstanding Resources Attraction?” Ecological Entomology 46: 552–561.

[ece370766-bib-0033] González‐Alvarado, A. , and F. Z. Vaz‐de‐Mello . 2021. “Towards a Comprehensive Taxonomic Revision of the Neotropical Dung Beetle Subgenus *Deltochilum* (*Deltohyboma*) Lane, 1946 (Coleoptera: Scarabaeidae: Scarabaeinae): Division Into Species‐Groups.” PLoS One 16: e0244657. 33406525 10.1371/journal.pone.0244657 PMC7787713

[ece370766-bib-0034] Greenacre, M. 2016. “Data Reporting and Visualization in Ecology.” Polar Biology 39: 2189–2205.

[ece370766-bib-0035] Halffter, G. , and W. D. Edmonds . 1982. The Nesting Behavior of Dung Beetles (Scarabaeinae): An Ecological and Evolutive Approach. México, D.F: Instituto de Ecología.

[ece370766-bib-0036] Halffter, G. , and V. Halffter . 2009. “Why and Where Coprophagous Beetles (Coleoptera: Scarabaeinae) eat Seeds, Fruits or Vegetable Detritus.” Boletín Sociedad Entomológica Aragonesa 45: 1–22.

[ece370766-bib-0037] Halffter, G. , and E. G. Matthews . 1966. “The Natural History of Dung Beetles of the Subfamily Scarabaeinae (Coleoptera: Scarabaeidae).” Folia Entomologica Mexicana 12: 1–312.

[ece370766-bib-0038] Hanski, I. , and Y. Cambefort . 1991. Dung Beetle Ecology. Princeton, USA: Princeton University Press.

[ece370766-bib-0039] Heinrich, B. , and G. A. Bartholomew . 1979. “The Ecology of the African Dung Beetle.” Scientific American 241: 146–157.

[ece370766-bib-0040] Hill, J. E. , T. L. Devault , J. C. Beasley , and O. E. Rhodes Jr. 2018. “Effects of Vulture Exclusion on Carrion Consumption by Facultative Scavengers.” Ecology and Evolution 8: 2518–2526. 29531672 10.1002/ece3.3840 PMC5838040

[ece370766-bib-0041] Hill, M. O. 1973. “Diversity and Evenness: A Unifying Notation and Its Consequences.” Ecology 54: 427–432.

[ece370766-bib-0042] Holter, P. , and C. H. Scholtz . 2007. “What Do Dung Beetles Eat?” Ecological Entomology 32: 690–697.

[ece370766-bib-0043] Hsieh, T. C. , K. Ma , and A. Chao . 2016. “iNEXT: An R Package for Rarefaction and Extrapolation of Species Diversity (H Ill Numbers).” Methods in Ecology and Evolution 7: 1451–1456.

[ece370766-bib-0044] Iannuzzi, L. , R. P. Salomão , F. C. Costa , and C. N. Liberal . 2016. “Environmental Patterns and Daily Activity of Dung Beetles (Coleoptera: Scarabaeidae) in the Atlantic Rainforest of Brazil.” Entomotropica 31: 196–207.

[ece370766-bib-0045] INMET . 2022. Instituto Nacional de Meteorologia. Graficos climatologicos, Amazonas, Manaus. https://clima.inmet.gov.br/GraficosClimatologicos/AM/82331.

[ece370766-bib-0046] Jost, L. 2006. “Entropy and Diversity.” Oikos 113: 363–375.

[ece370766-bib-0047] Junk, W. J. , J. Schöngart , M. T. F. Piedade , G. M. Soares , and F. Wittmann . 2020. “The Várzeas of the Brazilian Amazon River: Living With the Flood Pulse.” In River Culture: Life as a Dance to the Rhytm of the Waters, edited by K. M. Wantzen , 469–495. Paris, France: UNESCO.

[ece370766-bib-0048] Krell, F. T. , S. Krell‐Westerwalbesloh , I. Weiß , P. Eggleton , and K. E. Linsenmair . 2003. “Spatial Separation of Afrotropical Dung Beetle Guilds: A Trade‐Off Between Competitive Superiority and Energetic Constraints (Coleoptera: Scarabaeidae).” Ecography 26: 210–222.

[ece370766-bib-0049] Larsen, T. H. , and A. Forsyth . 2005. “Trap Spacing and Transect Design for Dung Beetle Biodiversity Studies 1.” Biotropica 37: 322–325.

[ece370766-bib-0050] Latty, T. , and J. S. Trueblood . 2020. “How Do Insects Choose Flowers? A Review of Multi‐Attribute Flower Choice and Decoy Effects in Flower‐Visiting Insects.” Journal of Animal Ecology 89: 2750–2762. 32961583 10.1111/1365-2656.13347

[ece370766-bib-0051] Liberal, C. N. , Â. M. I. de Farias , M. V. Meiado , B. K. Filgueiras , and L. Iannuzzi . 2011. “How Habitat Change and Rainfall Affect Dung Beetle Diversity in Caatinga, a Brazilian Semi‐Arid Ecosystem.” Journal of Insect Science 11: 114. 22224924 10.1673/031.011.11401 PMC3281362

[ece370766-bib-0052] Lira, L. A. , L. M. S. Aguiar Silvieira , and M. R. Frizzas . 2020. “Vertebrate Scavengers Alter the Chronology of Carcass Decay.” Austral Ecology 45: 1103–1109.

[ece370766-bib-0053] Louzada, J. N. C. , and P. R. C. Silva . 2009. “Utilisation of Introduced Brazilian Pastures Ecosystems by Native Dung Beetles: Diversity Patterns and Resource Use.” Insect Conservation and Diversity 2: 45–52.

[ece370766-bib-0054] Mayer, A. C. G. , and S. D. Vasconcelos . 2013. “Necrophagous Beetles Associated With Carcasses in a Semi‐Arid Environment in Northeastern Brazil: Implications for Forensic Entomology.” Forensic Science International 226: 41–45. 23398925 10.1016/j.forsciint.2012.11.019

[ece370766-bib-0055] McGeoch, M. A. , B. J. Van Rensburg , and A. Botes . 2002. “The Verification and Application of Bioindicators: A Case Study of Dung Beetles in a Savanna Ecosystem.” Journal of Applied Ecology 39: 661–672.

[ece370766-bib-0056] Mora‐Aguilar, E. F. , A. Arriaga‐Jiménez , C. M. Correa , et al. 2023. “Toward a Standardized Methodology for Sampling Dung Beetles (Coleoptera: Scarabaeinae) in the Neotropics: A Critical Review.” Frontiers in Ecology and Evolution 11: 1096208.

[ece370766-bib-0058] Oksanen, J. , G. L. Simpson , F. G. Blanchet , et al. 2020. “Vegan: Community Ecology Package.” https://cran.r‐project.org/web/packages/vegan/index.html.

[ece370766-bib-0059] Puker, A. , M. J. G. Oliveira , G. C. Silva , et al. 2024. “Structure of Dung Beetle Assemblages (Coleoptera: Scarabaeidae: Scarabaeinae) in Native Forest and Exotic Pastures in the Southwest of the Brazilian Amazon.” Biologia 79: 879–891.

[ece370766-bib-0060] R Core Team . 2022. R: A Language and Environment for Statistical Computing. Vienna, Austria: R Foundation for Statistical Computing.

[ece370766-bib-0061] Ratcliffe, B. C. 2013. “The Dung‐and Carrion‐Feeding Scarabs (Coleoptera: Scarabaeoidea) of an Amazonian Blackwater Rainforest: Results of a Continuous, 56‐Week, Baited‐Pitfalltrap Study.” Coleopterists Bulletin 67: 481–520.

[ece370766-bib-0062] Sakai, S. , and T. Inoue . 1999. “A New Pollination System: Dung‐Beetle Pollination Discovered in *Orchidantha inouei* (Lowiaceae, Zingiberales) in Sarawak, Malaysia.” American Journal of Botany 86: 56–61. 21680345

[ece370766-bib-0063] Salomão, R. P. , L. V.‐B. M. P. Cerqueira , A. A. C. Gomes , et al. 2022. “Dung or Carrion? Sex and Age Determine Resource Attraction in Dung Beetles.” Ecological Entomology 47: 52–62.

[ece370766-bib-0064] Salomão, R. P. , C. M. D. A. Correa , S. Santorelli Junior , et al. 2023. “Species Diet and the Effect of Different Spatial Bait Distribution on Assemblage of Dung Beetles in Amazonian White‐Sand Forest.” International Journal of Tropical Insect Science 43: 1153–1162.

[ece370766-bib-0065] Salomão, R. P. , D. de Alcântra Pires , F. B. Baccaro , et al. 2022. “Water Table Level and Soil Texture Are Important Drivers of Dung Beetle Diversity in Amazonian Lowland Forests.” Applied Soil Ecology 170: 104260.

[ece370766-bib-0066] Salomão, R. P. , and L. Iannuzzi . 2017. “How Do Regeneration Stages of Caatinga Forests Influence the Structure of Dung Beetle (Coleoptera: Scarabaeidae) Assemblage?” Coleopterists Bulletin 71: 578–588.

[ece370766-bib-0067] Schallhart, N. , M. J. Tusch , C. Wallinger , K. Staudacher , and M. Traugott . 2012. “Effects of Plant Identity and Diversity on the Dietary Choice of a Soil‐Living Insect Herbivore.” Ecology 92: 2650–2657. 10.1890/11-2067.1 23431595

[ece370766-bib-0068] Scholtz, C. H. , A. L. V. Davis , and U. Kryger . 2009. Evolutionary Biology and Conservation of Dung Beetles, 1–567. Sofia‐Moscow: Pensoft.

[ece370766-bib-0069] Selva, N. , B. Jędrzejewska , W. Jędrzejewski , and A. Wajrak . 2005. “Factors Affecting Carcass Use by a Guild of Scavengers in European Temperate Woodland.” Canadian Journal of Zoology 83: 1590–1601.

[ece370766-bib-0070] Silva, P. G. D. , and M. I. M. Hernández . 2015. “Spatial Patterns of Movement of Dung Beetle Species in a Tropical Forest Suggest a New Trap Spacing for Dung Beetle Biodiversity Studies.” PLoS One 10: e0126112. 25938506 10.1371/journal.pone.0126112 PMC4418735

[ece370766-bib-0072] Stavert, J. R. , B. A. Drayton , J. R. Beggs , and A. C. Gaskett . 2014. “The Volatile Organic Compounds of Introduced and Native Dung and Carrion and Their Role in Dung Beetle Foraging Behaviour.” Ecological Entomology 39: 556–565.

[ece370766-bib-0073] Stone, R. L. , E. L. Engasser , and M. L. Jameson . 2021. “Heads or Tails? Dung Beetle (Coleoptera: Scarabaeidae: Scarabaeinae and Aphodiinae) Attraction to Carrion.” Environmental Entomology 50: 615–621. 33751102 10.1093/ee/nvab012

[ece370766-bib-0074] Vaz‐De‐Mello, F. Z. 2008. “Synopsis of the New Subtribe Scatimina (Coleoptera: Scarabaeidae: Scarabaeinae: Ateuchini), with Descriptions of Twelve New Genera and Review of *Genieridium*, New Genus.” Zootaxa 1955: 1–75. 10.11646/zootaxa.1955.1.1.

[ece370766-bib-0075] Wardhaugh, C. W. , N. E. Stork , and W. Edwards . 2012. “Feeding Guild Structure of Beetles on Australian Tropical Rainforest Trees Reflects Microhabitat Resource Availability.” Journal of Animal Ecology 81: 1086–1094. 22530991 10.1111/j.1365-2656.2012.01988.x

[ece370766-bib-0076] Zuur, A. F. , E. N. Ieno , N. J. Walker , A. A. Saveliev , and G. M. Smith . 2009. Mixed Effects Models and Extensions in Ecology With R, 1–574. New York: Springer.

