# Supplementary material for: The Most Attractive Is Not Always the Preferred: Lessons From Necrophagous Dung Beetle Assemblages in a Region of the Central Amazon
Source: Ecol Evol. 2024 Dec 23;14(12):e70766. doi: 10.1002/ece3.70766 (PMC11664420; doi:10.1002/ece3.70766)
Supplement: Supplementary file 1 — Data S1. [file ECE3-14-e70766-s001.docx]

**SUPPLEMENTARY MATERIAL**

**FIGURE S1.** Scheme of food attractiveness. Each set of traps was distant 150 m from each other.

**
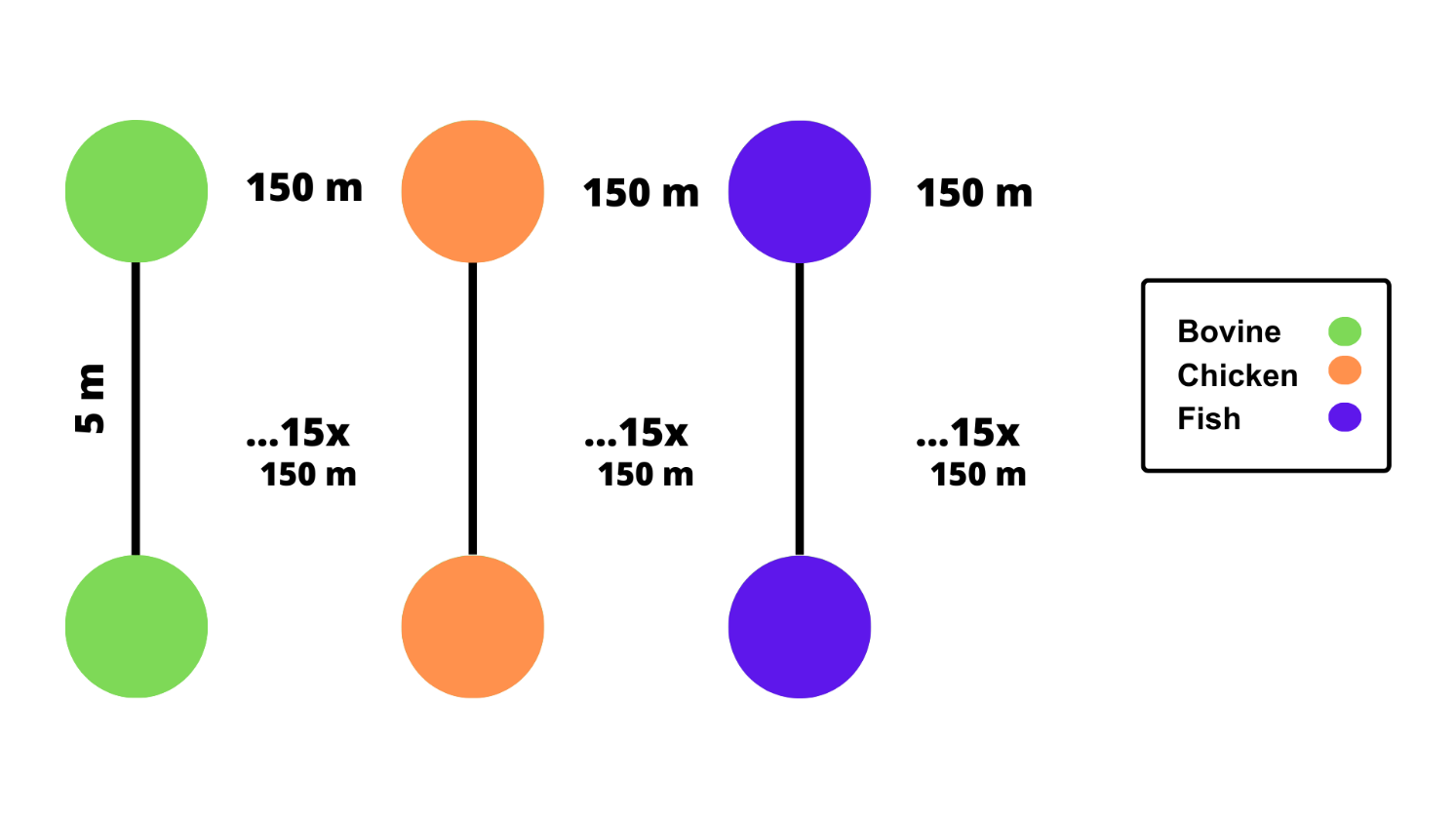
**

**FIGURE S2.** Scheme of food preference experiments. Each set of traps was distant 150 m from each other, and trap types were randomly distributed.


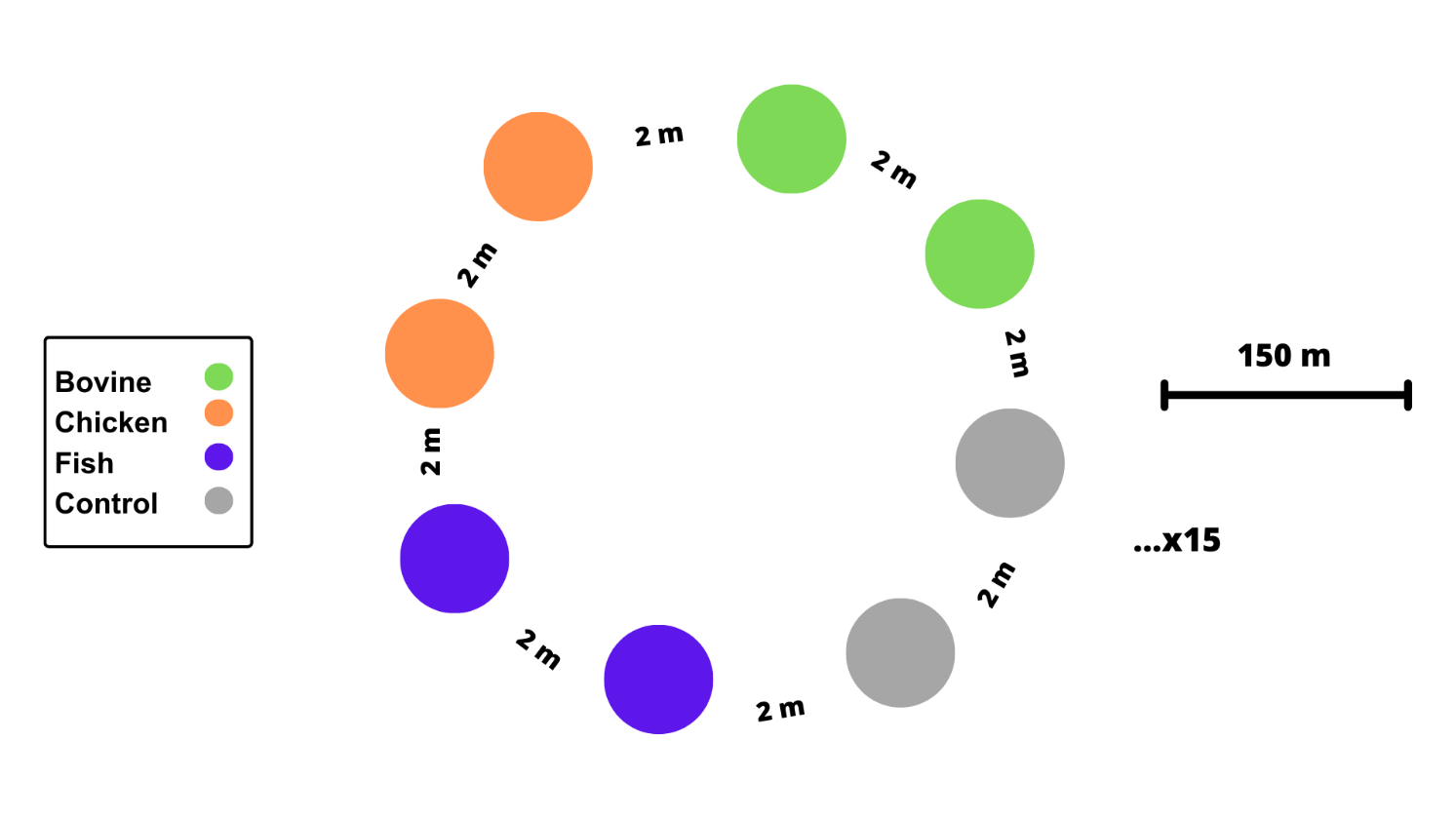


**Table S1**. PERMANOVA and PERMDISP pairwise test to examine differences in species composition and differences in multivariate dispersion among three baits for the food attractiveness and food preference experiments in the Brazilian Amazon.

|  | Food attractiveness | | | | |
| --- | --- | --- | --- | --- | --- |
| Group | PERMANOVA | |  | PERMDISP | |
|  | t value | p value |  | t value | p value |
| Bovine versus Chicken | 3.01 | 0.001 |  | 5.89 | 0.001 |
| Bovine versus Fish | 3.01 | 0.001 |  | 1.70 | 0.115 |
| Chicken versus Fish | 2.48 | 0.001 |  | 3.31 | 0.001 |
|  | Food preference | | | | |
| Group | PERMANOVA | |  | PERMDISP | |
|  | t value | p value |  | t value | p value |
| Bovine versus Chicken | 1.98 | 0.002 |  | 0.90 | 0.452 |
| Bovine versus Fish | 2.49 | 0.001 |  | 0.84 | 0.444 |
| Chicken versus Fish | 0.91 | 0.555 |  | 0.17 | 0.871 |
